# Sodium thiosulphate for recalcitrant dystrophic calcinosis cutis ‒ An effective but tricky treatment option^؆^

**DOI:** 10.1016/j.abd.2025.501222

**Published:** 2025-11-01

**Authors:** André Aparício Martins, André Pinho

**Affiliations:** Dermatology and Venereology Department, Unidade Local de Saúde de Coimbra, Coimbra, Portugal

Dear Editor,

Calcinosis cutis (CC) is the deposition of insoluble calcium salts in the skin and subcutaneous tissue.^1^ Dystrophic CC is the most common subtype. Caused by local injury, it is frequently associated with connective tissue diseases. Although rare, dystrophic CC affects 18%‒49% of systemic sclerosis (SSc) patients.^2^ CC has a high morbidity due to pain, functional impairment, ulceration, and secondary infections, leading to disability.^3^ Lacking large-scale controlled trials, dystrophic CC remains without standard therapy. Intralesional sodium thiosulfate (STS) was proposed as a therapeutic option based on case reports and small case series.

A 50-year-old woman with limited SSc, evolving over 19-years, referred stiffness, pain and ulceration on the first four digital pulps of each hand for one year. She underwent surgical excision of calcium deposits of the 2^nd^ and 3^rd^ fingers bilaterally and started diltiazem 90 mg/day. For two years, there was symptomatic progression. Skin involvement also included sclerodactyly and malar cutaneous telangiectasia. In addition, her SSc was complicated by oesophageal dysmotility, partially controlled with proton pump inhibitors, and severe Raynaud phenomenon (RF), under bosentan. No evidence of pulmonary arterial hypertension or other systemic involvement. Laboratory tests were positive for Antinuclear antibodies (ANA) and anticentromere antibodies (ACA). Physical examination revealed painful, stiff, skin-coloured papules, 4‒10 mm, distributed on the pulp of the 1^st^, 2^nd^ and 4^th^ right fingers and the 1^st^ left finger, compatible with CC (Fig. 1), as confirmed by hand radiography (Fig. 2). Intralesional STS treatment was proposed due to significant morbidity, progression under diltiazem and the involved site. The procedure was performed using an aseptic technique and under digital nerve block, with a 250 mg/mL solution. Depending on the size of the lesion and pulp distensibility, 0.1‒1 mL of STS solution was injected into the base of the calcifications. Initially, at a 6 to 8-week interval, and after improvement, every 3-months. 25 sessions were performed in 5-years, with continuous symptomatic improvement and better functional status. Hand radiography confirmed a sustained reduction in calcification size (Fig. 3). During the first 2-years, episodes of ulceration and spontaneous drainage of liquified calcium deposits were reported, both self-limited. Additionally, 2 local infections were treated with oral antibiotics. There were no systemic adverse events or analytical abnormalities. However, 2 weeks after the last administration, the patient developed necrosis on the pulp of the 1^st^ right and 2^nd^ left fingers. Treatment with bosentan was suspended 3-months earlier due to long-term control of RF. Oedema from STS infiltration may have triggered a more pronounced RF episode, leading to pulp necrosis. The patient underwent treatment with iloprost 0.4 ng/Kg/min for 3-days and resumed bosentan, with complete healing. Currently, she is asymptomatic, with residual calcifications in hand radiography.

**Figure 1:**
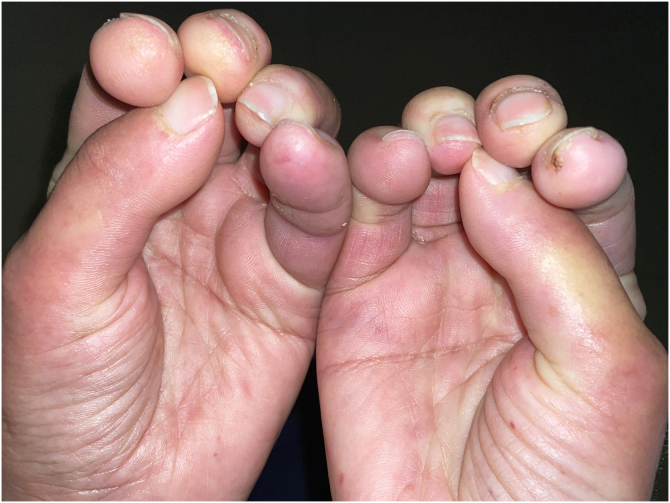
Skin-coloured papules, distributed on the pulp of fingers of both hands.

**Figure 2:**
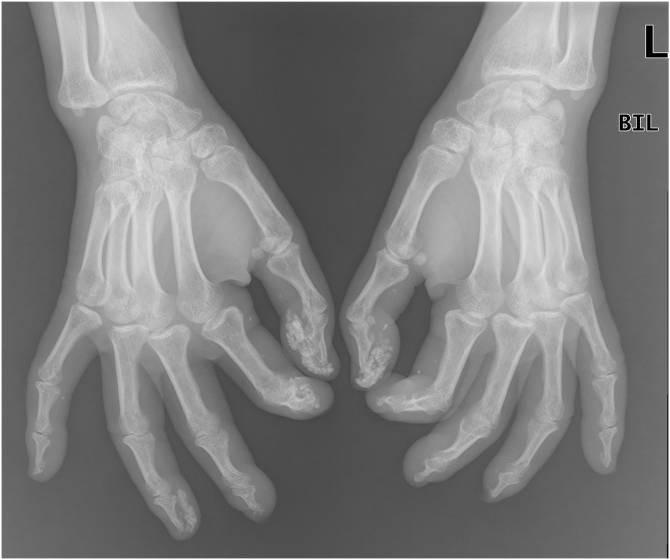
Hands simple radiography (oblique view) before treatment with intralesional sodium thiosulfate.

**Figure 3:**
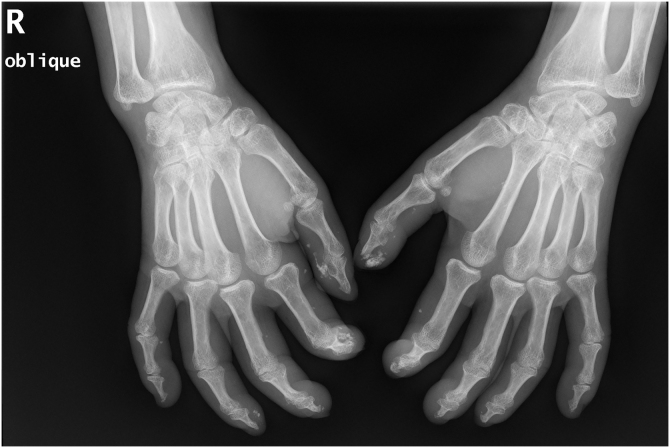
Hands simple radiography (oblique view) after 25 sessions of treatment with intralesional sodium thiosulfate.

In dystrophic CC, chronic cellular damage and hypoxia can trigger ectopic calcification with normal serum calcium levels.^4^ CC usually presents as firm, painful plaques or nodules, associated with functional impairment.^3^ Lesions can ulcerate, fistulize and become infected. In patients with SSc, extensor surfaces, palms and soles are commonly involved.^4^ Pharmacological therapy is the first-line, mainly high-dose calcium-channel inhibitors, bisphosphonates, colchicine and minocycline.^5^ Surgery is reserved for small symptomatic lesions as primary treatment or an adjunct to drug therapy.^6^ Successful treatment with intravenous, topical and intralesional STS has been reported in a small number of patients. STS is a chelating agent that increases calcium solubility up to 100.000 times, in addition to anti-inflammatory, vasodilative and anti-oxidative effects.^7^ Intravenous administration has adverse events, such as headaches, vomiting, hypotension, hypernatremia and metabolic acidosis, limiting its use in systemic diseases.^8^ Topical STS is not associated with systemic adverse events but is slow, requires strict compliance and may inadequately penetrate the skin, being recommended on small lesions.^1^, ^7^

Intralesional STS provides local delivery to deep lesions without systemic adverse effects. The concentration of 250 mg/mL is the most frequently described.^4^ The volume (0.1‒1 mL) and the interval between administrations (weekly to 3-months) vary according to lesions’ size, patient tolerance and clinical evolution.^1^, ^4^, ^7^ Intralesional STS has a rapid onset and complete remission is described in 50%‒100% of patients.^4^ Transient local pain and secondary infections are the most frequently reported adverse events.^8^ Additionally, it may be advantageous in SSc patients with thickened skin.^8^

In conclusion, intralesional STS seems a promising option in recalcitrant, severe CC. The safety profile reinforces its applicability, but patient selection is mandatory due to a long treatment course. In addition, RF treatment is essential, as the vascular compression due to the intralesional administration may predispose to digital ischemic necrosis. Randomized controlled trials are needed to improve the optimal dosage, administration technique, and minimize the risk of pulp necrosis.

## Financial support

None declared.

## Authors' contributions

André Aparício Martins: Writing of the manuscript or critical review of important intellectual content; Intellectual participation in the propaedeutic and/or therapeutic conduct of the studied case; Critical review of the literature; Final approval of the final version of the manuscript.

André Pinho: Writing of the manuscript or critical review of important intellectual content; Intellectual participation in the propaedeutic and/or therapeutic conduct of the studied case; Critical review of the literature; Final approval of the final version of the manuscript.

## Research data availability

Does not apply.

## Conflicts of interest

None declared.
